# Real-Time Monitoring of Biotinylated Molecules Detection Dynamics in Nanoporous Anodic Alumina for Bio-Sensing

**DOI:** 10.3390/nano9030478

**Published:** 2019-03-23

**Authors:** Laura Pol, Chris Eckstein, Laura K. Acosta, Elisabet Xifré-Pérez, Josep Ferré-Borrull, Lluis F. Marsal

**Affiliations:** Departament d’Enginyeria Electrònica, Elèctrica i Automàtica, ETSE, Universitat Rovira i Virgili, Avda. Països Catalans 26, 43007 Tarragona, Spain; laura.pol@urv.cat (L.P.); chris.eckstein@urv.cat (C.E.); laurakaren.acosta@urv.cat (L.K.A.); elisabet.xifre@urv.cat (E.X.-P.)

**Keywords:** nanoporous anodic alumina, streptavidin, biotin, RIfS, biosensing

## Abstract

The chemical modification, or functionalization, of the surfaces of nanomaterials is a key step to achieve biosensors with the best sensitivity and selectivity. The surface modification of biosensors usually comprises several modification steps that have to be optimized. Real-time monitoring of all the reactions taking place during such modification steps can be a highly helpful tool for optimization. In this work, we propose nanoporous anodic alumina (NAA) functionalized with the streptavidin-biotin complex as a platform towards label-free biosensors. Using reflective interferometric spectroscopy (RIfS), the streptavidin-biotin complex formation, using biotinylated thrombin as a molecule model, was monitored in real-time. The study compared the performance of different NAA pore sizes in order to achieve the highest response. Furthermore, the optimal streptavidin concentration that enabled the efficient detection of the biotinylated thrombin attachment was estimated. Finally, the ability of the NAA-RIfS system to quantify the concentration of biotinylated thrombin was evaluated. This study provides an optimized characterization method to monitor the chemical reactions that take place during the biotinylated molecules attachment within the NAA pores.

## 1. Introduction

In the last few decades, many different classes of biosensors for biomedical applications have received great attention [1,2,3]. The principles of a biosensor consist of a bio-receptor (antibodies, aptamers), a transducer, and a signal processor. In practice, a transitional chemical interface is commonly introduced between the transducer and bio-receptor to promote the access of the target to the bioreceptor. Such a transitional chemical interface comprises chemical molecules with convenient functional groups, whose purpose is to facilitate the attachment of the bioreceptor in the most efficient manner.

Optical biosensors are based on the interaction of light with a biorecognition element. There are two principal categories of optical biosensors: label-based and label-free sensors. Label-based sensors rely on using labeled molecules that produce a colorimetric, fluorescent, or luminescent signal whereas in label-free sensors the signal is directly generated by the interaction of the biomolecules with the surface [4,5]. Labeling assays only provide endpoint read outs, and do not allow continuous monitoring. Furthermore, labeling assays need exhaustive washing processes, and the labeling molecules can interfere with the interaction of key molecules during the bioreception process because they can block access to the binding sites.

On the other hand, in optical label-free sensors, it is possible to monitor continuous binding events in real-time. Several label-free biosensor techniques are reported in the literature. For instance, surface plasmon resonance (SPR) [6,7], localized surface plasmon resonance (LSPR) [8,9,10], ellipsometry [11], surface-enhanced Raman spectroscopy (SERS) [12]. In particular, the reflectance interference spectroscopy (RIfS) technique has demonstrated its capability to monitor biomolecule interactions in real-time with simple and cost-effective procedure in porous silicon and nanoporous anodic alumina (NAA) [13] with proteins [14,15], antibodies [16,17], aptamers [18], or cells [19]. 

Nanoporous anodic alumina has been used as a promising biosensor platform in the last few years [20,21]. NAA is a material with straight pores growing perpendicular to the surface with a cost-effective fabrication process that provides a well-defined and controllable geometry [22]. Furthermore, NAA can be easily modified chemically and provides a biocompatible platform with high surface-to-volume ratio with chemical resistance, thermal stability, and hardness [23]. The surface chemistry of NAA permits the introduction of useful functional groups (e.g., carboxyl, amin) by means of self-assembly silanization with aminopropyl-triethoxy silane (APTES) [24], mercaptopropyl-triethoxy silane [25], 3-isocyanatopropyl triethoxy silane (ICN) [26], polyethylene glycol-silane (PEG-silane) [27], and several other silanes. Moreover, NAA possesses unique optical properties, such as characteristic reflectance spectra governed by Fabry–Perot interferences, that exploit NAA as a platform to study the chemical interactions in biosensors using RIfS [28,29].

On account of its suitable properties, streptavidin is a protein widely used in the transitional chemical interface to facilitate the access of the bioreceptor to the target [25]. It is reported that the avidin-biotin interaction is one of the strongest non-covalent interactions in nature. Streptavidin is an analog of avidin that is extracted from *Streptomyces avidinii* with a molecular weight of 58 kDa [30]. Streptavidin is a tetrameric protein able to bind with four biotins with a dissociation constant of 10^−15^ M [31]. It is reported that streptavidin is a protein with a long half-life (around 50 days) [32] and is one of the most thermally stable proteins. Furthermore, the streptavidin-biotin complex can persist stably for 35 h [33]. Biotin is also a very stable molecule and easy to bind covalently to most of the biomolecules, and a very wide range of commercial biotinylated molecules for biosensor applications can be found. Several studies report positive results on the streptavidin-biotin system in NAA to check covalent attachment [34] or to immobilize cell proteins [19]. Thrombin is a plasmatic protein involved in blood coagulation processes with a well-known structure and properties [35]. Thrombin has been studied as a target model in several biosensor approaches such as electrochemical [36,37], colorimetric [38], and fluorescence [39,40] biosensors.

Because of the properties described above, NAA with streptavidin in the transitional chemical interface can be a very versatile and stable platform that permits the achievement of specific and sensitive biosensors. In this work, we study the transitional chemical interface formation using RIfS in real-time, as well as the further attachment of a biotinylated model molecule, which in this case is thrombin. With this, it is possible to obtain information about the dynamics of the binding of streptavidin to the silanized surface of NAA. Furthermore, we show that RIfS permits to monitor in real-time and to quantify the attachment process of the biotinylated molecule to the transitional chemical interface. In addition, this work also permits to study the sensitivity of an NAA platform to the detection of biotinylated thrombin.

## 2. Materials and Methods

### 2.1. Materials

Aluminum foils of 99.999% of purity and 0.5 mm of thickness were purchased from Goodfellow Ltd. (Cambridge, UK). Oxalic acid (H_2_C_2_O_4_), phosphoric acid (H_3_PO_4_), perchloric acid (HClO_4_), chromium oxide (CrO_2_), copper chloride (CuCl_2_), ethanol (C_2_H_5_OH), acetone ((CH_3_)_2_CO), 2-(N-Morpholino)ethanesulfonic acid (MES), phosphate buffered saline (PBS), bovine serum albumin (BSA), phosphate buffered saline with Tween™ 20 (PBS-T), streptavidin, N-Hydroxysuccinimide (NHS), N-(3-Dimethylaminopropyl)-N′ethylcarbodiimide hydrochloride (EDC), (3-Aminopropyl)triethoxysilane (APTES), and thrombin were purchased from Merck KGaA (Darmstadt, Germany)Biotinylated thrombin was purchased from Thermo Fisher Scientific (Madrid, Spain). Double-deionized (DI) water (18.6 MΩ, PURELAB Option-Q) was used for all solutions.

### 2.2. NAA Fabrication

NAA samples were fabricated by the well-known two-step anodization method [41] with 0.3 M of oxalic acid at 40 V and 5 °C following the protocol described in the literature [42]. The first step was carried out during 20 h; this first formed alumina layer was removed during 3 h in an etching solution of H_3_PO_4_ 6% wt. and H_2_CrO_7_ 1.8% wt. at 70 °C. The second step was carried out until a total charge of Q = 20 C per sample was used in the anodization. The area of one sample was 1.7 cm^2^. In this way, samples with an NAA layer with 5 μm depth pores and 30 nm pore diameter were obtained. The pore diameter was then adjusted to 50 nm and 60 nm by immersion on NAA in H_3_PO_4_ at 35 °C during 10 and 20 min respectively. These values of pore diameter are the lowest and highest that give a measurable signal in the RIfS procedure. Pore radii smaller than 50 nm hinders the diffusion of the molecules into the pores, while pore radii bigger than 60 nm result in a layer too porous to create the light interferences necessary for the RIfS technique. Figure S1 in the supplementary information shows two top-view SEM pictures of samples with 50 nm and 60 nm pore diameter (a and b), respectively, and a cross-section image of the sample with a pore diameter of 50 nm (c). The images show that pore sizes have a small dispersion in diameter and that pores grow uniformly along its length.

### 2.3. Sample Preparation

**Figure 1:**
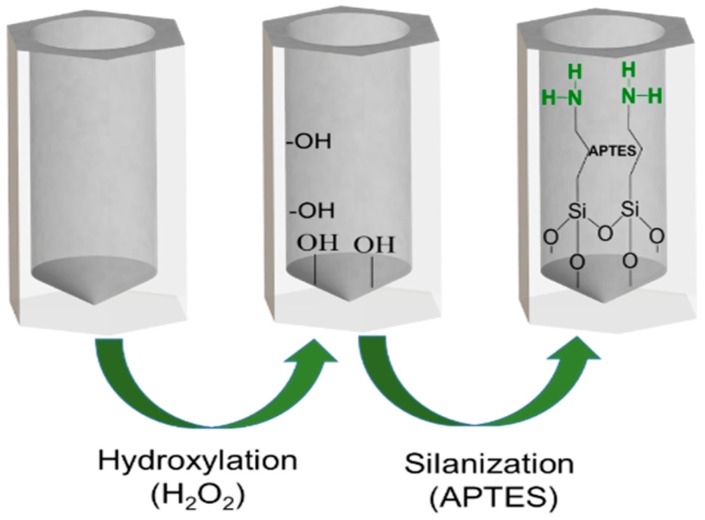
Schematics of the steps for the immobilization of APTES in NAA.

Before the real-time interferometric measurements, the samples were chemically pretreated by immobilization of APTES, as shown in Figure 1, in the NAA surface. Firstly, the NAA was hydroxylated by immersion in boiling hydrogen peroxide (H_2_O_2_) as reported in previous works [34] for 1 h with continuous stirring followed by a washing step with ethanol and dried under a nitrogen flux. Then NAA was incubated for 1 h in a solution with 15 mL of anhydrous toluene and 1.5 mL of APTES with continuous stirring under nitrogen flux. Next, the NAA were sonicated in a toluene bath for 5 min to remove remaining APTES. Finally, the NAA with APTES was washed again with ethanol, dried under nitrogen flux, and placed in the oven at 110 °C overnight to promote silane cross-linking. Just before the streptavidin-biotininylated thrombin binding experiments, a thin film of 10 nm gold was deposited onto the NAA by sputtering.

### 2.4. Reflectometric Interference Spectroscopy System (RIfS)

**Figure 2:**
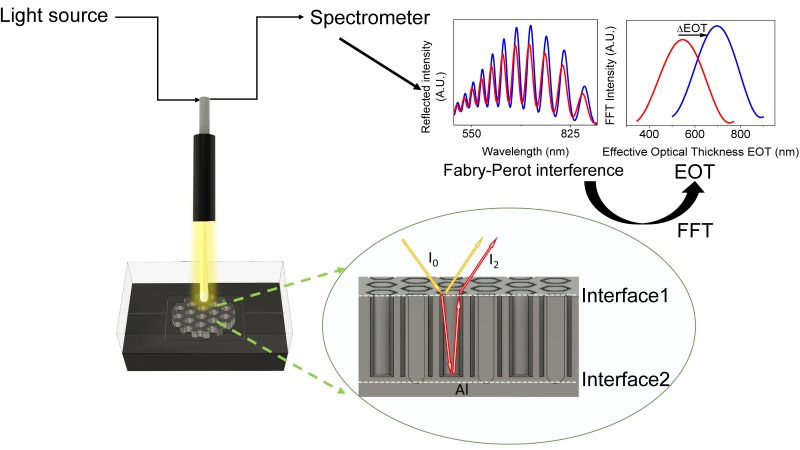
Schematic of the reflectance interferometric spectroscopy and flow cell system.

RIfS is a technique based on the measurement of the changes in the effective refractive index of NAA thin films when molecules attach to the inner pore surface. The basics of RIfS detection in flow cell are illustrated in Figure 2. NAA was placed in a transparent flow cell system which permitted the flow of different solutions with a rate of 500 μL/min and its diffusion into the pores. The total volume of the solutions (streptavidin and biotinylated thrombin) was 1 mL and they were recirculated in the flow cell during all the infiltration steps. Previous experiments with glucose solutions demonstrated that molecules diffuse fast and completely along the pores [29]. After diffusion, molecules can attach to the inner pore surface contributing to the change in effective refractive index. Simultaneously, white light is directed to the NAA surface and the reflected light was collected by a spectrometer which registered an interferometric reflectance spectrum. Interferences arose from the optical path difference between the two beams reflected at the top and bottom interfaces of the NAA film as in a Fabry–Perot interferometer. Since the light reflected at the bottom traveled across the pore, the optical path difference was directly proportional to the effective refractive index of the NAA layer, constituted by the oxide, the medium filling the pores and the molecular layers deposited on the inner pore surfaces. The interferometric reflectance spectrum showed oscillations that were analyzed by a Fourier transform procedure that allowed us to estimate the optical path thickness which could then be related to the effective optical thickness (EOT, defined as the physical thickness times the effective refractive index) of the NAA layer. The spectrometer measured one spectrum every 10 s and the Fourier transform was calculated by the same computer controlling the spectrometer using a custom-made MATLAB script. This allowed us to observe the changes in the EOT in real-time.

### 2.5. Real-Time Monitoring of Biotinylated Thrombin Attachment to Streptavidin into NAA Pores

**Figure 3:**
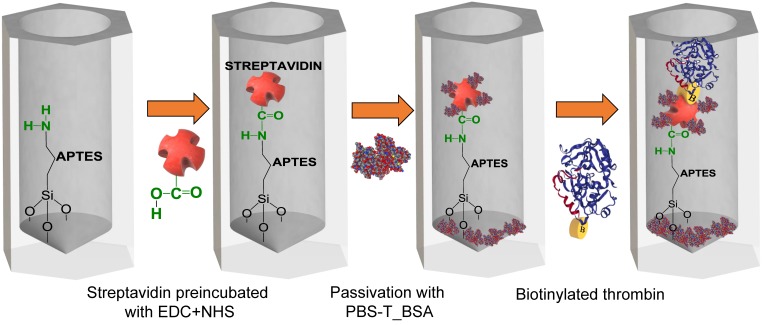
Schematics of the different NAA modification steps in the real-time monitoring of biotinylated thrombin attachment experiments.

In each of the binding experiments, one pretreated NAA sample with APTES was placed in the flow cell system to monitor the biotinylated thrombin attachment in NAA in real-time, as shown in Figure 3. Before each experiment, a preparation step was applied consisting of flowing PBS until a steady value of EOT registered. Simultaneously to this preparation step, streptavidin was incubated for 20 min with EDC and NHS in a proportion of 1:10:25, respectively, to activate the carboxyl groups of streptavidin.

The first step in the experiment was the injection of this activated streptavidin solution in the flow cell. The instant at which the activated streptavidin solution is injected in the flow cell is defined as the initial time (t = 0 s) and the EOT at that instant is taken as the reference EOT, EOT_0_. In this first step, the free amino group of APTES (–NH_3_) formed a peptide bond with the carboxyl group of streptavidin (–COOH). This first step was applied until a stable EOT value was observed. After this, PBS was flowed to remove any non-specifically attached streptavidin molecules. 

The second step consisted of the injection of 3% BSA with 0.05% Tween 20, pH 7.4 (PBS-T_BSA) to block the remaining sites on the NAA pore surface and the free carboxyl groups of streptavidin that did not attach to the amino groups of APTES, which could lead to non-specific bindings. To end this second step, a flow of PBS was introduced to wash unbound remaining molecules.

The third and final step in the experiments was to flow the biotinylated thrombin solution. As in the previous steps, a wash with PBS was introduced to finish, and the variation in EOT was registered. The total EOT variation in this experiment was designated as ΔEOT.

## 3. Results and Discussion

### 3.1. Study of the Influence of the Pore Diameter in the Detection of the Biotinylated Thrombin

**Figure 4:**
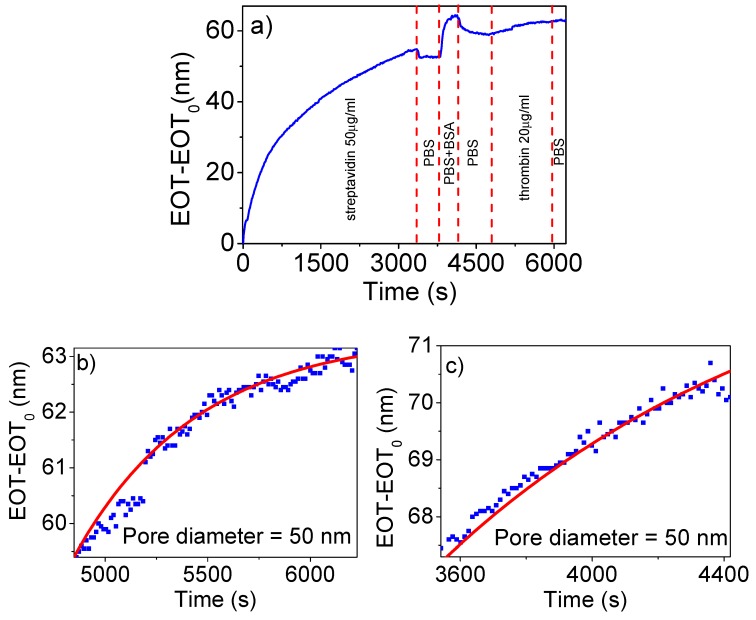

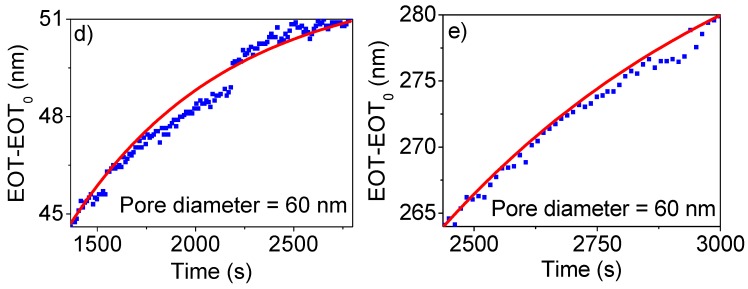
(**a**) Example of the registered change in EOT as a function of time for one of the performed biotinylated thrombin attachment experiments. (**b**,**c**) A close-up corresponding to the biotinylated thrombin step for two experiments corresponding to NAA with 50 nm pore diameter. (**d**,**e**) A close-up for the same step for two experiments corresponding to NAA with 60 nm pore diameter.

The first goal of this work was to evaluate the ability of RIfS to detect the binding of a biotinylated molecule to streptavidin that is grafted on the pore walls of NAA and the influence of the pore size in the event of detection. Figure 4a shows a characteristic example of the evolution of EOT along the streptavidin-biotinylated thrombin attachment experiment. The data correspond to an NAA with 60 nm diameter pores and to a concentration of streptavidin in the first step of 50 µg/mL. The dashed lines indicate the starting time for each step in the experiment. First, an increase in EOT is observed as streptavidin is attached. Such an increase shows three different stages: the first one with a bigger slope, followed by a smaller rate of growth, and reaching a steady value at the end. When a steady EOT value is reached, PBS is flowed to remove any non-specifically attached streptavidin molecules, resulting in a sudden decrease in EOT of 2.4 nm, which corresponds to a 4.4% decrease of the value obtained at the end of the streptavidin step.

The next step consists of passivation with 3% bovine serum albumin in the PBS-T which results in a fast increase of 10 nm EOT followed by a steady value for 192 s. After this, again PBS is flowed to clean any non-attached BSA with a reduction of EOT as a consequence. It has to be noted that if the BSA passivation step and the subsequent PBS cleaning are considered as a whole, the EOT reaches a value greater than that achieved by the previous streptavidin step. 

Next, biotinylated thrombin at a concentration of 20 µg/mL is flowed through the cell for 1285 s. A slow and steady increase in EOT is observed with a final value of 3.6 nm above the initial one. Finally, after this step, a flow of PBS is introduced which does not produce any noticeable change in the EOT.

The increase in EOT observed at the streptavidin step, and the small decrease with the subsequent PBS cleaning step are consistent with the attachment of the streptavidin molecule to the APTES on the NAA pore surface and with the removal of the non-bound molecules with the PBS flow. The increase and stable value of the signal at the BSA passivation stage can be explained by the fact that BSA is occupying the free spaces between the APTES molecules on the NAA pore surfaces. The higher value of EOT after such a step indicates that a certain amount of BSA remains attached to the pore walls, actually passivating the NAA surface and the carboxyl groups in the streptavidin. Finally, the increase in EOT in the biotinylated thrombin binding step certifies the ability of the RIfS method to detect such a binding event.

The graphs in Figure 4b,c show the evolution of EOT corresponding to the last step in the experiment (biotinylated thrombin binding) for two different NAA with 50 nm diameter pores, while Figure 4d,e correspond to NAA with 60 nm diameter pores. The plots include a best-fit curve with exponential dependence:

$$EOT-EOT_{0,th}=A\cdot \left(1-e^{-\frac{t-t_{0,th}}{B}}\right)$$

where t_0,th_ corresponds to the time of introduction of the biotinylated thrombin solution, EOT_0,th_ corresponds to the EOT value at that instant, A and B are the adjusted parameters of amplitude and reaction time. With the best fit, the values of A and B can be used to obtain a robust estimate of the total variation of EOT in this step, designated as ΔEOT.

In all cases, a steady increase of EOT with time is observed and the increase is correlated with the pore diameter: the estimated average increase for 50 nm diameter is 3 nm, while the average increase for 60 nm diameter is 11 nm. The complete evolution of the EOT with time for such experiments is shown in Figure S1 of the supplementary material. This result indicates that a bigger pore diameter improves the detectability of the binding of biotinylated molecules onto streptavidin-functionalized NAA.

### 3.2. Influence of the Streptavidin Concentration in the First Step

**Figure 5:**
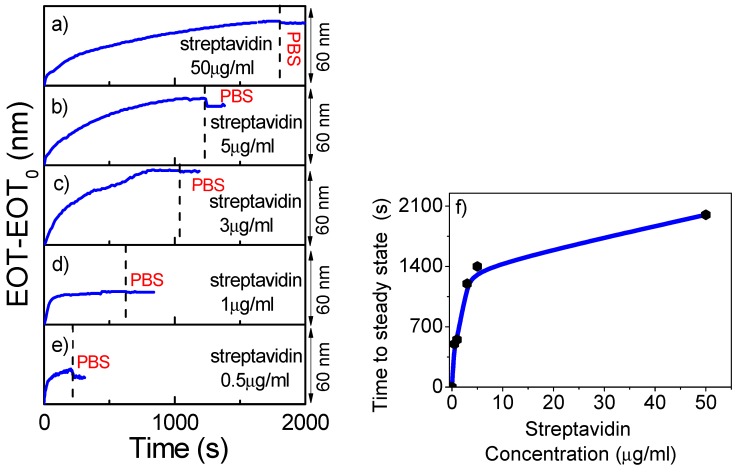
Variation of EOT against time for the streptavidin attachment step for different streptavidin concentrations, indicated in the plots. (**a**) 50 µg/mL, (**b**) 5 µg/mL, (**c**) 3 µg/mL, (**d**) 1 µg/mL, and (**e**) 0.5 µg/mL. (**f**) Time to reach the steady-state against streptavidin concentration.

It has been noticed that the change of EOT over time in the streptavidin binding step shows two different stages, an initial one at a high increase rate, followed by another stage at a smaller increase rate. In order to understand the reasons for such behavior, a study was conducted to test the coverage of the NAA pores with streptavidin, for different streptavidin concentrations. The study was carried out for 60 nm pore diameter NAA and for decreasing streptavidin concentrations from 50 µg/mL and down to 0.5 µg/mL. Figure 5 shows the results of this study, where graphs a) to e) plot the change in EOT for the streptavidin binding step at concentrations of 50 µg/mL, 5 µg/mL, 3 µg/mL, 1 µg/mL, and 0.5 µg/mL, respectively. 

It was observed that the flow of the streptavidin solution resulted in a clear increase in EOT for all studied concentrations. The graphs a) to e) can be classified into two main groups: for the 50, 5 and 3 µg/mL concentrations, two different stages in the EOT variation can be identified; a first one in which a fast increase in EOT is observed until an inflection point is reached in a short time (60 s for 50 µg/mL, 157 s for 5 µg/mL and 408 s for 3 µg/mL), followed by a second stage with a slower increase in rate. This second stage reaches a steady EOT value after a given time, at which PBS is introduced in the flow cell to remove all the non-bound molecules (the start of this washing step is indicated by the vertical dashed lines in the graphs a) to e) of Figure 5). However, for the 1 µg/mL and 0.5 µg/mL concentrations, only one stage is recognized: a fast increase in EOT which reaches a stable value in a short time that is not followed by a second increase.

The time at which a stable EOT value is reached is defined as the time at which the increased rate in EOT is smaller than 0.1 nm/s for 10 consecutive measured spectra (this is taken over a 100-second period). Such an instance is indicated in the graphs in Figure 5a–e with a vertical dashed line. The estimated times to reach a stable EOT are: 1465 s for 50 µg/mL, 1009 s for 5 µg/mL, 792 s for 3 µg/mL, 444 s for 1 µg/mL, and 192 s for 0,1 µg /ml. Figure 5f plots these times as a function of the concentration. The graph shows clearly the existence of two different behaviors in the streptavidin binding process: a one-stage for low concentrations and a two-stage for high concentrations. It was also observed that at the very low concentration (0.5 µg/mL), a clear stable EOT signal was not obtained.

These two different ranges in the EOT changes with streptavidin concentration indicate that at high concentrations there are two different processes in the streptavidin attachment to the NAA pore walls. In the first stage, the activated carboxyl group of streptavidin (–COOH) is bounded to the amino group of the APTES (–NH_2_). Then, if the concentration of streptavidin is high, a second process occurs consisting of streptavidin-streptavidin aggregation as the activated carboxyl group of one streptavidin can bind with the amino group of another streptavidin.

### 3.3. Influence of the Biotinylated Thrombin Concentration

**Figure 6:**
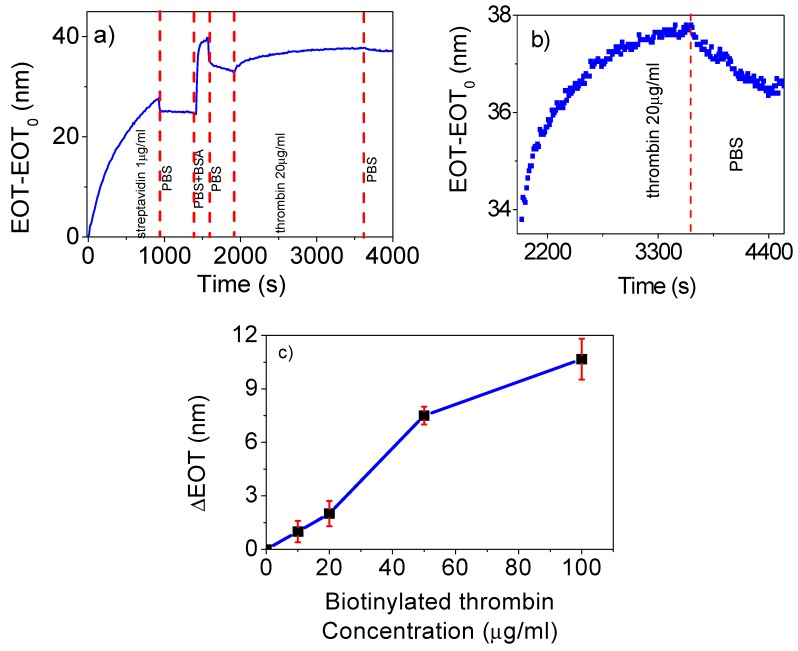
(**a**) Example of EOT variation with time for a biotinylated thrombin attachment experiment intended to evaluate the sensitivity of the NAA platform, corresponding to the concentrations specified on the graph. (**b**) A close-up of the EOT variation with time for one of the experiments conducted at a biotinylated thrombin concentration of 20 µg/mL. (**c**) Total change of EOT in the biotinylated thrombin attachment step (EOT) against biotinylated thrombin concentration.

To quantify the capabilities of the RIfS technique to detect the binding event of biotinylated molecules to NAA pore surfaces covered with streptavidin, different binding experiments were conducted for increasing biotinylated thrombin concentrations. The results of such experiments are shown in Figure 6. On the basis of the previous results, these experiments were carried for a fixed concentration of streptavidin at 1 µg/mL and for a 60 nm pore diameter. Figure 6a shows an example of the entire experiment: first we flowed PBS until a steady baseline EOT value was achieved. At t = 0 s, streptavidin (1 µg/mL) was introduced until a stable EOT was reached, at which point PBS was flowed to remove any unbound molecules. Next, the BSA solution is flowed to passivate the surface, followed by a subsequent PBS flow again to remove the non-bound BSA. The final step was the flow of the biotinylated thrombin solution at 20 µg/mL followed by a final PBS flow in order to wash all the non-binding molecules. 

The introduction of streptavidin resulted in a rapid increase in the EOT signal, which became stable after 927 s in a single stage, providing a monolayer of streptavidin on the NAA pore surface. The washing with PBS produced a very small decrease in EOT consistent with the removal of any unbound molecules. The introduction of the BSA solution resulted in an increase of EOT corresponding to the electrostatic binding of the BSA to the NAA pore surface not previously covered with streptavidin. Subsequently, a decrease with the PBS-T flow was observed, down to an EOT above the value after the streptavidin step. This confirmed the effect of the passivation. Finally, when biotinylated thrombin was flowed, another increase in EOT was observed and a stable EOT value was reached after 1213 s. The level of EOT remained the same after the final wash step with PBS. 

Figure 6b shows a close-up of the change in EOT corresponding to the biotinylated thrombin binding step for the experiment in Figure 6a. The graph shows that a clear increase in EOT can be observed until a stable EOT value is reached. For all the conducted experiments, the EOT level after the final PBS washing step is over to the EOT before the biotinylated thrombin addiction (Figure S3 supplementary material). The change in EOT in this last step after the PBS for this thrombin concentration leads to an ΔEOT of 2.4 nm.

Figure 6c shows a plot of the ΔEOT for the biotinylated thrombin concentration. Experiments were performed for the 10, 20, 50, and 100 µg/mL concentrations in triplicates for each concentration. The reported value corresponds to the average of the three replicas, while error bars correspond to the maximum and minimum measured ΔEOT. The blue lines connecting the experimentally measured points are included as a guide for the eye. The average ΔEOT for each concentration are: 0.16 nm for of 10 µg/mL, 2 nm for 20 µg/mL, and 7.5 nm for 50 µg/mL, and with 10.6 nm for 100 µg/mL. The graph indicates that thrombin can be clearly detected for concentrations above 10 µg/mL and that EOT response begins to saturate above 100 µg/mL. 

## 4. Conclusions

In this study, we demonstrate the ability of nanoporous anodic alumina-based reflection interferometric spectroscopy in the flow cell to monitor in real-time the attachment of thrombin through the streptavidin-biotin system. The results introduced in this work are proof that the streptavidin-biotin complex in NAA is a suitable platform towards RIfS-based biosensors.

We evaluated the influence of pore size diameter of NAA in the EOT response and estimated that the best value is around 60 nm. Smaller pores can hinder the binding processes due to the reduced amount of space, while bigger pores are not achievable by the NAA preparation procedures.

We assess the optimal concentration of streptavidin to obtain the best attachment of biotinylated thrombin. It was observed that, at concentrations above 1 µg/mL, aggregation between streptavidin molecules was produced. On the other hand, concentrations below 1 µg/mL of streptavidin were not sufficient to produce complete coverage of the inner pore surface and led to noisy values in the EOT signal. These results show that the optimal concentration of streptavidin is 1 µg/mL.

We monitored in real-time all the binding events from the silanized NAA from biotinylated thrombin attachment with special focus in the biotinylated thrombin binding event. This system can detect different concentrations of biotinylated thrombin. We prove that we can quantify biotinylated molecules at concentrations above 10 µg/mL. The results in this work pave the way to apply NAA as an optical biosensor suitable for several kinds of biomolecules.

## Supplementary Materials

The following are available online at https://www.mdpi.com/2079-4991/9/3/478/s1. Figure S1: SEM images of NAA, Figure S2: Registered change in EOT as a function of time in entire experiments for different pore diameter, Figure S3: Best replicates of all the experiments for different concentrations of biotinylated thrombin.
