# Supplementary material for: Real-Time Monitoring of Biotinylated Molecules Detection Dynamics in Nanoporous Anodic Alumina for Bio-Sensing
Source: Nanomaterials (Basel). 2019 Mar 23;9(3):478. doi: 10.3390/nano9030478 (PMC6474190; doi:10.3390/nano9030478)
Supplement: Supplementary file 1 [file nanomaterials-09-00478-s001.pdf]

SUPPLEMENTARY MATERIAL

Real-time Monitoring of Biotinylated Molecules Detection Dynamics in Nanoporous Anodic Alumina for Bio-sensing

Laura Pol, Chris Eckstein, Laura K. Acosta, Elisabet Xifré-Pérez, Josep Ferré-Borrull and Lluis F. Marsal*

UNIVERSITAT ROVIRA i VIRGILI

Universitat Rovira i Virgili

Group of Nano-electronic and Photonic Systems (NePhoS)

Departament d'Enginyeria Electrònica, Elèctrica i Automàtica,
Avda. Països Catalans 26, 43007 Tarragona, Spain

Figure S1: ESEM images of NAA a) 50 nm pore diameter and b) 60 nm pore diameter

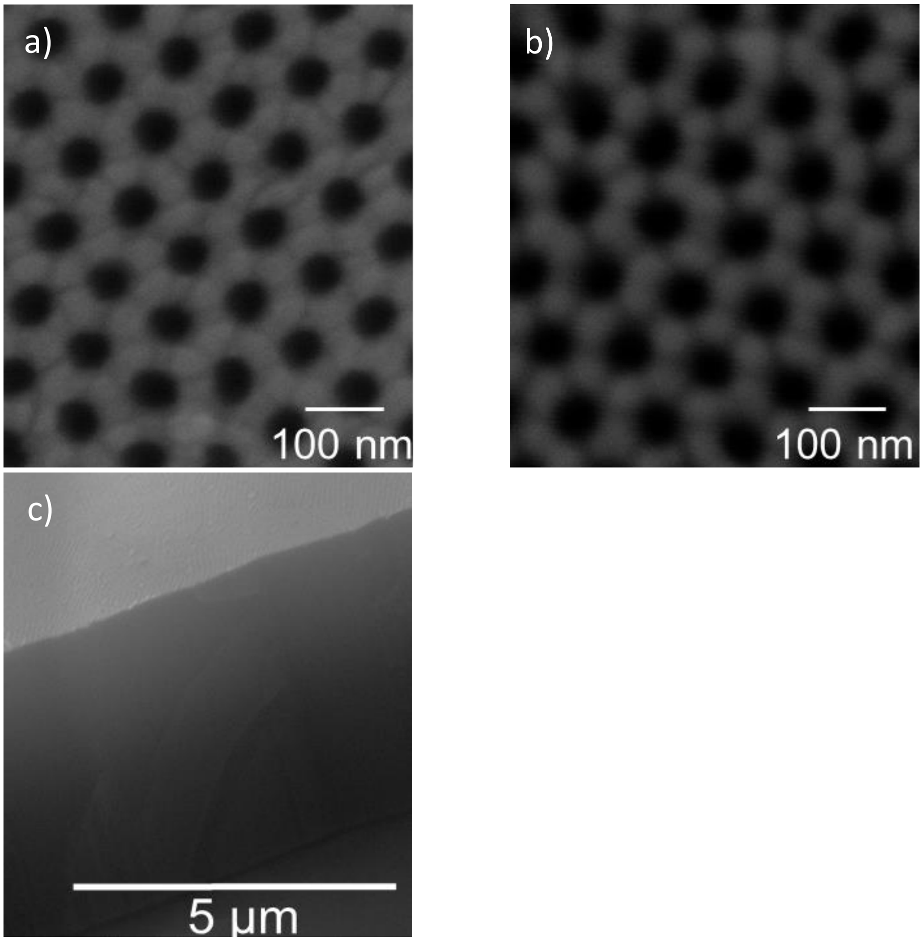

Figure S2: Registered change in EOT as a function of time for complete biotinylated thrombin attachment experiments for the different tested pore diameters. The different steps in the experiment are indicated in the graphs as numbers and correspond to: 1 = Streptavidin, 2 = PBS, 3 = PBS-T_BSA, 4 = PBS, 5 = Biotinylated thrombin, 6 = PBS. Figures a) and b) correspond to NAA with 50 nm pore diameter; figures c) and d) correspond to NAA with 60 nm pore diameter.

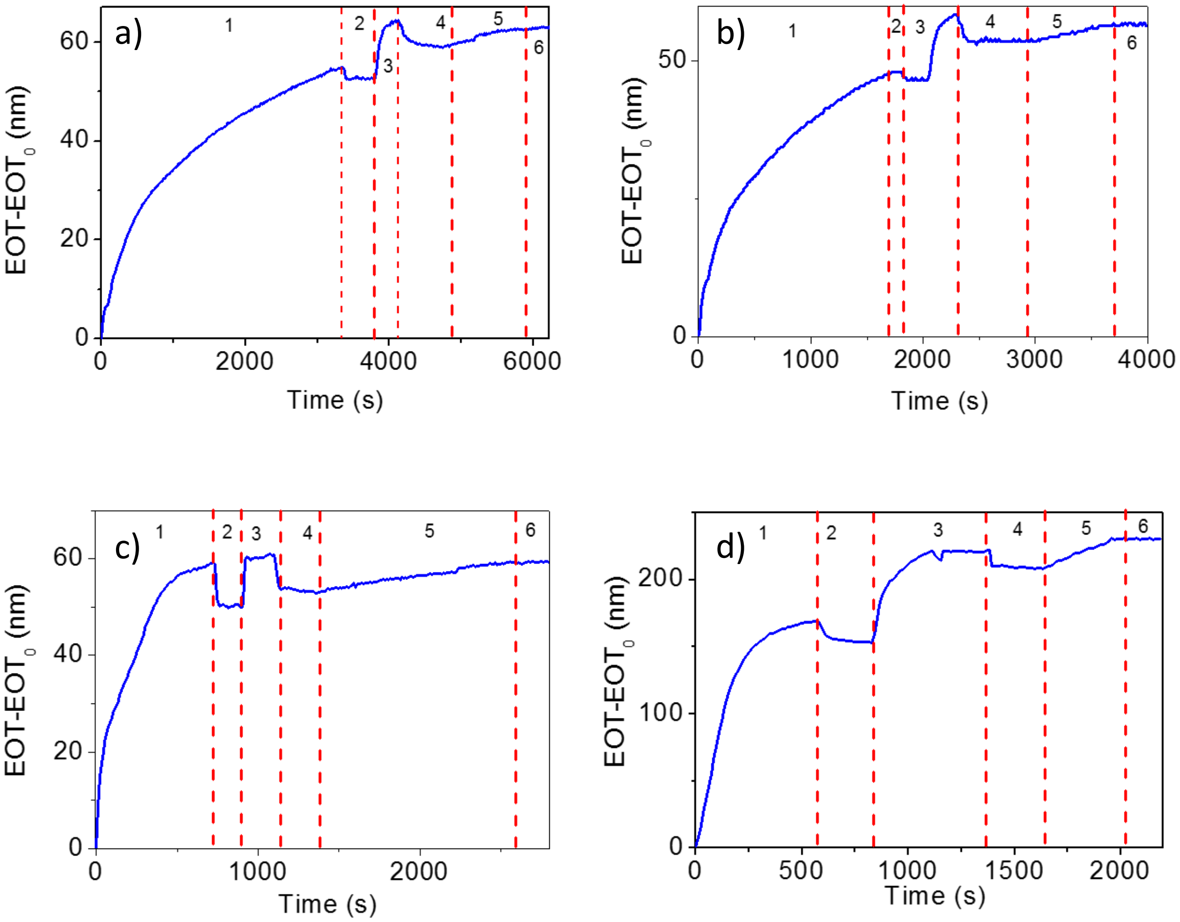

Figure S3: Registered change in EOT as a function of time for complete biotinylated thrombin attachment experiments for the different tested biotinylated thrombin concentrations. The different steps in the experiment are indicated in the graphs as numbers and correspond to:

1= Streptavidin, 2= PBS, 3= PBS-T_BSA, 4= PBS, 5= Biotinylated thrombin, 6= PBS. Figures a) and b) correspond to 100 μg/ml; figures c) and d) correspond to 50 μg/ml; figures correspond to e) and f) 20 μg/ml; figures g) and h) correspond to 10 μg/ml.

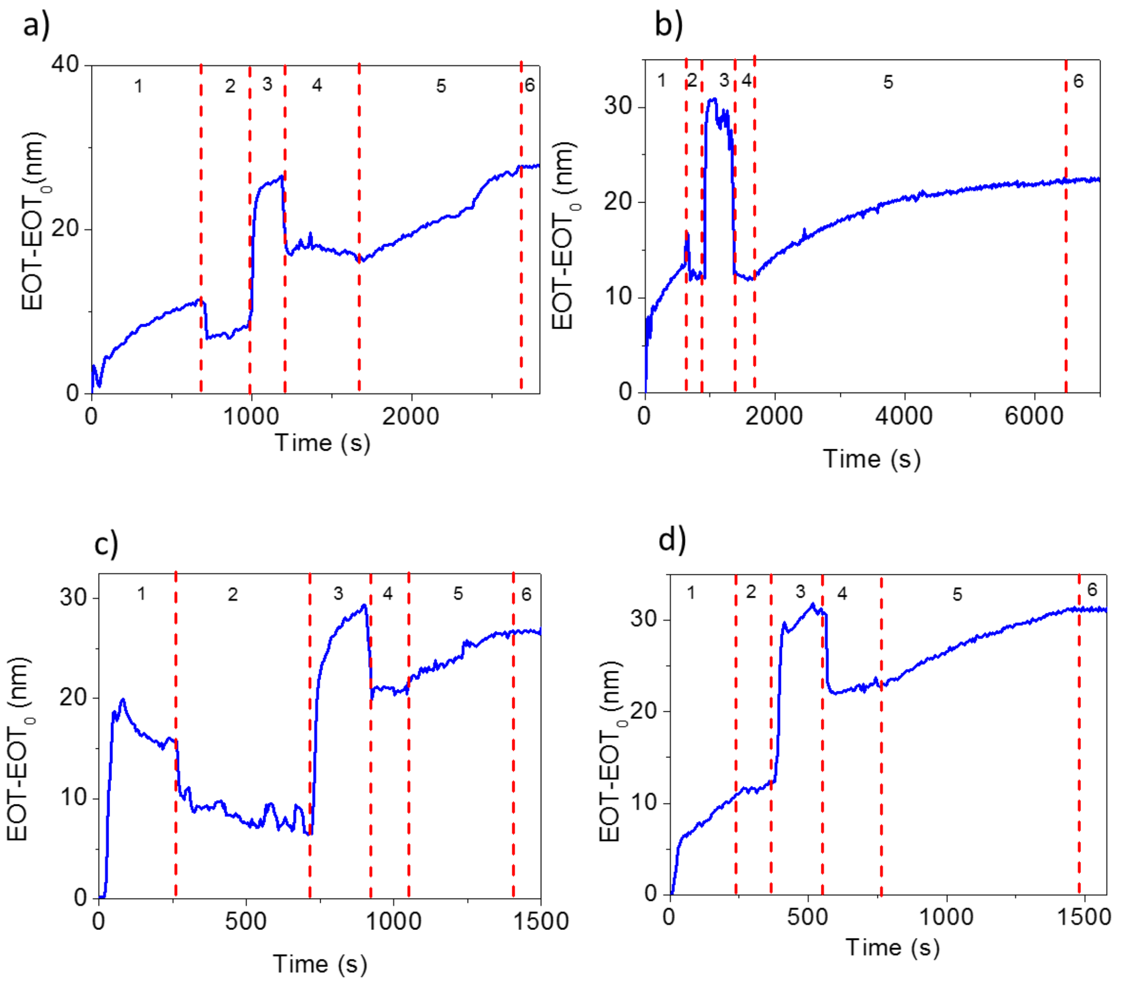

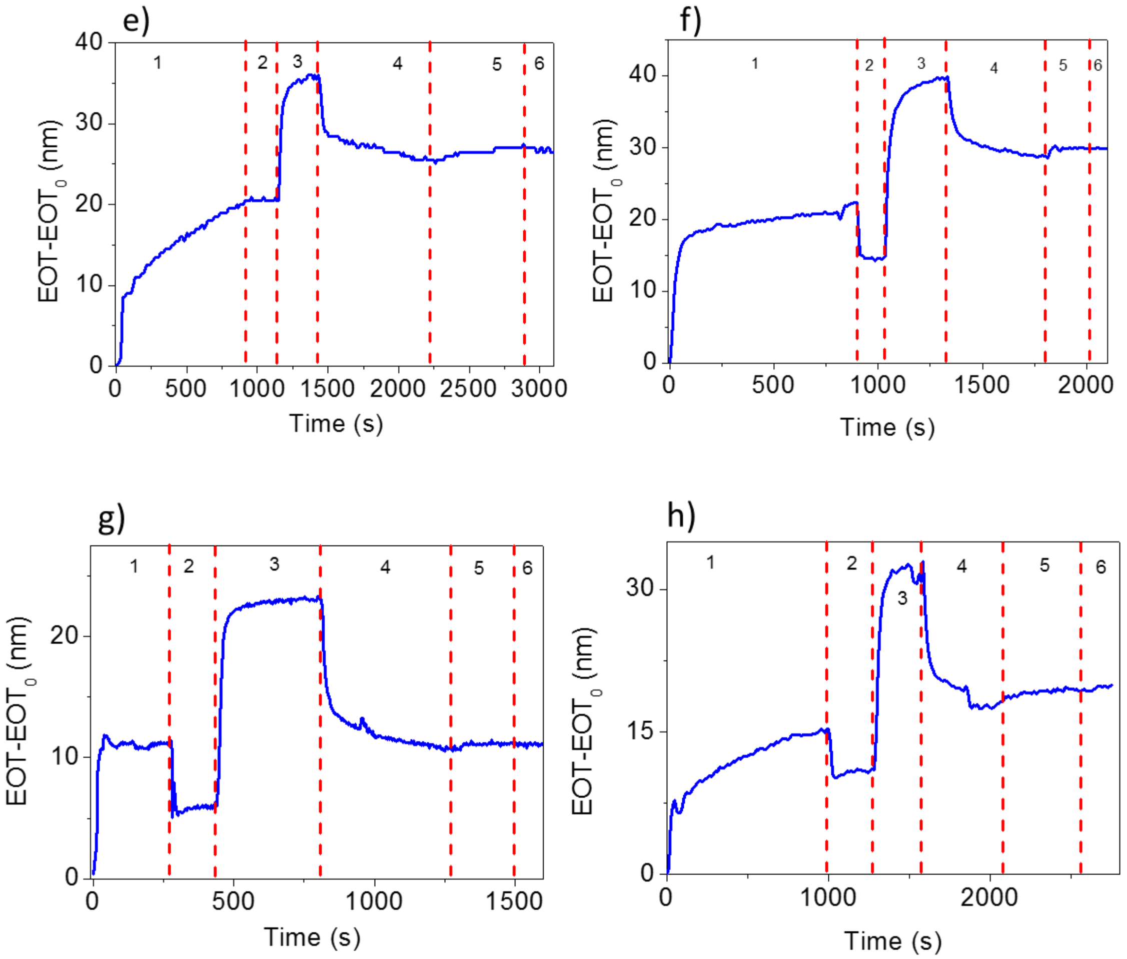
e)
f)
g)
h)
EOT-EOT$_0$ (nm)
Time (s)
1
2
3
4
5
6
